# Life-History Traits from Embryonic Development to Reproduction in the American Cockroach

**DOI:** 10.3390/insects13060551

**Published:** 2022-06-16

**Authors:** Liangguan Lin, Jiazhen Wen, Sheng Li, Fangfang Liu

**Affiliations:** 1 Guangdong Provincial Key Laboratory of Insect Developmental Biology and Applied Technology, Institute of Insect Science and Technology & School of Life Sciences, South China Normal University, Guangzhou 510631, China; amylin@m.scnu.edu.cn (L.L.); 2021023041@m.scnu.edu.cn (J.W.); lisheng@scnu.edu.cn (S.L.); 2 Guangdong Laboratory for Lingnan Modern Agriculture, Guangzhou 510631, China; 3 Guangmeiyuan R&D Center, Guangdong Provincial Key Laboratory of Insect Developmental Biology and Applied Technology, South China Normal University, Meizhou 514779, China

**Keywords:** cockroach, embryonic development, nymphal instars, secondary sexual characteristics, reproduction

## Abstract

**Simple Summary:**

The American cockroach is a widely distributed sanitary pest closely related to human life. The American cockroach is not only a hygienic pest that we all know but also beneficial to humans as its extract can be used medicinally and could be a model organism for physiology and neuroscience studies. In this study, we provide a life table of the American cockroach in a stable environment, including embryonic development, nymphal instars, and adult reproduction. Newly laid eggs hatch into nymphs after about 35 days of embryonic development. Under sufficient materials and space, gregarious nymphs undergo 14 molts before transforming into adults. Adult females can produce fertile offspring whether they have mated or not. On average, mated females produce an ootheca every 4 days, while unmated females produce an ootheca every 10 days. Each ootheca contains 12–16 eggs. Additionally, group living seems to improve the survival rate of offspring of unmated females.

**Abstract:**

The American cockroach, *Periplaneta americana* (Insecta: Blattodea: Solumblattodea: Blattidae), is an urban hygiene pest but also a model organism for physiology and neuroscience study. However, the current description of the developmental process of the American cockroach is insufficient. In this study, we provide a life table of the American cockroach in a stable environment, including embryonic development, nymphal instars and adult reproduction. Our results show that there are 14 nymphal instars of the American cockroach in groups with sufficient living materials and space. The secondary sexual characteristics are evident in last-instar nymphs and adults, namely, the complete absence of the anal stylus in females. The entire embryonic development process was divided into 20 stages on the basis of lateral-view observations of the embryos. The formation of the embryo involves the fusion of paired blastoderm regions with higher cellular density, similar to that in other insects of Polyneoptera. With respect to reproduction, the gamogenetic females produced their first ootheca earlier than the parthenogenic females, and the frequency of oviposition was higher for the former throughout adulthood. Interestingly, group living seems to improve the parthenogenesis success rate in the American cockroach.

## 1. Introduction

The American cockroach, *Periplaneta americana* (Insecta: Blattodea: Solumblattodea: Blattidae), is an urban hygiene pest widely distributed in warm and humid regions around the world [1,2] and is closely related to human activities. Its native habitat is thought to be located in Africa, but it was introduced to the Americas with human activities in the early 16th century and then gradually spread throughout the world. The American cockroach is a social insect with scototaxis and prefers to live in shady and moist environments, such as urban sewers with access to food sources [3]. Due to its living environment, the American cockroach carries a variety of pathogens and allergens that trigger allergic reactions and asthma in certain individuals [4,5,6,7,8,9,10].

This hemimetabolous insect has three life stages: the egg, nymph and adult. In adulthood, females can produce fertile offspring whether they mate or not [11,12], and all fertile progeny are diploid [13,14]. The difference is that the progeny of the virgins are all females, while approximately half of the progeny of mated females are males. The American cockroach lays 12 to 16 eggs each time. Mature eggs are expelled sequentially and encased individually with secretions produced by the colleterial glands, eventually forming an ootheca [15,16]. The developmental threshold temperature of eggs is 15.8 °C, reaching an effective accumulated temperature of 415.8 ± 38.5 degree-days before hatching [17]. Newly hatched first-instar nymphs must undergo 6–14 molts before emerging into adults [3]. The American cockroach was observed to have 9–14 nymphal stages when raised alone at room temperature [18,19]. This intraspecific variation in nymphal instar numbers is a common phenomenon in hemimetabolous insects and is influenced by environmental and genetic factors [20].

However, with the deepening of research on this species, these fragmentary descriptions remain far from sufficient. Previously, our laboratory reported the genomic information of the American cockroach and conducted development and reproduction functional studies [1]. We know that the American cockroach has the characteristics of rapid growth, variable molts, high fecundity, and remarkable tissue regeneration capability. However, we do not know the molecular mechanisms of these biological processes? These related researches will be very significant and interesting. Unfortunately, it is very inconvenient for us to uncover those mechanisms due to the lack of basic research data and the ambiguity of the developmental stages of the American cockroach. Thus, we conducted this basic experiment on the American cockroach. In this study, we observed three life stages of cockroaches: embryonic development, nymphal growth and adult reproduction. The entire embryonic development process was divided into 20 stages according to certain characteristics. We confirmed that 14 molts during the nymphal growth stage can be clearly distinguished by measuring three auxiliary parameters (body mass, body length and body width). We also observed reproductive efficiency of gamogenesis is higher than that of parthenogenesis. These results of the American cockroach can be beneficial to studying the development and physiology process and mechanisms.

## 2. Materials and Methods

### 2.1. Insects

The line of *P. americana* was provided by Dr. Huiling Hao (Shanghai), whose line has been maintained with inbreeding for 30 years. The *P. americana* was maintained under laboratory conditions as previously described [1,16,21]. Previous studies have shown that the optimum temperature for the growth and reproduction of *P. americana* is 28–30 °C [17,22,23]. Therefore, the cockroaches in this study (the same as mentioned in reference [17,22,23]) were reared at 29 °C and 70 ± 5% relative humidity (RH) under a 12:12 h (light: dark) photoperiod in a plastic box (45 cm × 32 cm × 27 cm) and were fed sufficient water and commercial rat food with 1.06% calcium and 0.99% phosphate.

### 2.2. Fixation, Staining, and Observation of Embryos and the Germ Band

All embryos were fixed and stained at room temperature (20–30 °C) on a shaker. The ootheca (days 1–7) was treated with saturated sodium hypochlorite solution for approximately 1 h, carefully punctured 2 or 3 times with tweezers in PBS and fixed with 4% paraformaldehyde for more than 3 days. Subsequently, the embryos were dissected from the ootheca in PBS. Additionally, the germ band (days 4–9) was dissected from the partially fixed embryo, which was treated with 4% paraformaldehyde for approximately 2 days.

The ootheca (day 8–35) was heated at 80 °C for 10 min in a water bath. Then, the oothecal capsule was carefully torn away, and the batch of embryos was fixed with 4% paraformaldehyde for more than 2 days. Subsequently, the embryos were dissected from the egg membrane.

Fixed embryos and germ bands were stained using a DNA-specific fluorescent dye, 4′,6-diamidino-2-phenylindole dihydrochloride (DAPI, Sigma D5492-5MG, diluted with 1 mL of ddH_2_O and then to approximately 10 μg/mL with PBS), for 24 h or several days. Images of stained embryos and germ bands were captured with an Olympus FluoView FV3000 confocal microscope and analyzed with FV31S-SW software (Olympus, Tokyo, Japan).

### 2.3. Observation of Nymph Growth and Adult Reproduction

Newly hatched first-instar nymphs were collected within 24 h and group-housed. Referring to the modeling method of *Blattella asahinai* for estimating nymphal instars [24], the body mass, length, and width of the nymphs were measured as three indicators. Cockroaches were massed on an analytical balance (Sartorius) after anaesthetizing with CO_2_ to measure the weight of each cockroach. Precision can be measured to three decimal places. Cockroach body length was measured from head to the end of the abdomen and width was the widest part of the tergum. Each molting time of the nymphs was recorded from hatching to adulthood, and 10 individuals were randomly selected to measure body weight, length, and width on the first day after molting. Newly emerged adults were collected from the laboratory colony and housed alone, in pairs, or a group. When cockroaches were group-housed, each group contained at least 30 individuals. All oothecae produced by every female were collected and recorded. The animals were observed every morning and evening.

Images of the insects and oothecae were captured with a Nikon DS-Ri2 camera and a Nikon SMZ25 microscope.

### 2.4. Data Analysis

We used GraphPad Prism 8 software, and the statistical analyses were performed with Student’s *t*-test or one-way ANOVA. The significance values were calculated by one-way ANOVA. The values are shown as the mean ± standard deviation.

## 3. Results

### 3.1. Embryonic Development

The embryonic development period of American cockroaches was approximately 35 days under incubation at 29 °C. Based on the changes in embryonic features and referring to the embryonic development processes of *Blattella germanica* [25] and *Eucorydia yasumatsui* [26], the embryonic period of the American cockroach was divided into 20 stages.

**Figure 1:**
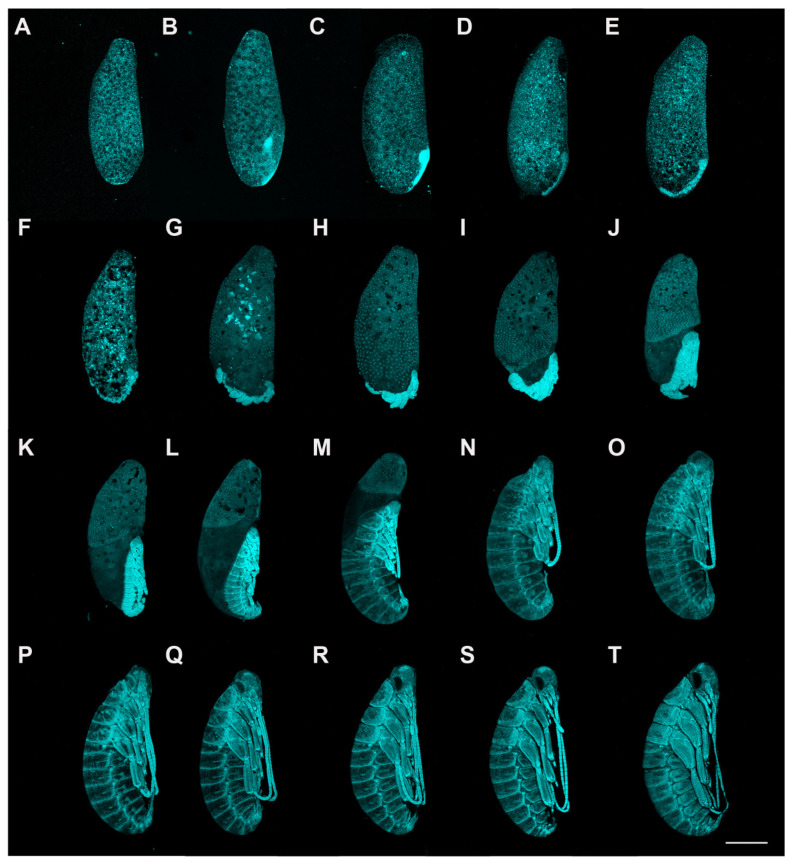
Embryonic development of *P. americana* (overview). Lateral views, ventral to the right. Fluorescence images with DAPI-stained nuclei are shown. (**A**) Stage 1. (**B**) Stage 2. (**C**) Stage 3. (**D**) Stage 4. (**E**) Stage 5. (**F**) Stage 6. (**G**) Stage 7. (**H**) Stage 8. (**I**) Stage 9. (**J**) Stage 10. (**K**) Stage 11. (**L**) Stage 12. (**M**) Stage 13. (**N**) Stage 14. (**O**) Stage 15. (**P**) Stage 16. (**Q**) Stage 17. (**R**) Stage 18. (**S**) Stage 19. (**T**) Stage 20. Scale bars: 1000 μm.

**Figure 2:**
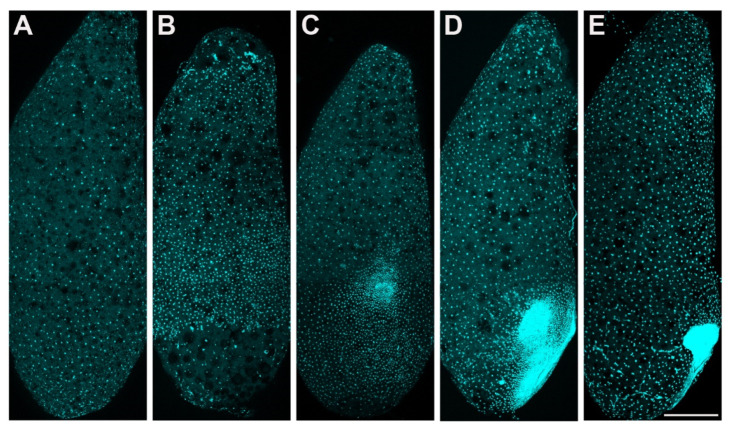
*P. americana* embryos in stage 1 and stage 2. Lateral views, ventral to the right. (**A**,**B**) Syncytial blastocyst stage. (**A**) Early-stage 1, approximately 24 h. (**B**) Late-stage 1, approximately 48 h. (**C**–**E**) Stage 2, the formation of the embryo. (**C**) Early-stage 2, approximately 72 h. (**D**) Middle stage 2, approximately 84 h. (**E**) Late-stage 2, approximately 96 h. Scale bars: 500 μm. Schemes follow the same formatting.

Stage 1. Days 0–2 (Figure 1A and Figure 2A,B): Syncytial blastocyst stage. In this stage, the embryo is a syncytial blastoderm with hundreds to thousands of nuclei, and all the cleavage nuclei are contained within a common cytoplasm. Initially, the zygote nucleus undergoes several mitotic divisions within the inner portion of the egg. After approximately one day, some of the divided nuclei migrate to the egg periphery.

Stage 2. Day 3 (Figure 1B and Figure 2C–E): With the progressive proliferation of cells, a pair of lateral regions with higher cellular density begin to differentiate on the posterior ventral side of the egg. These regions migrate medially and then condense to the ventroposterior region of the egg, assuming a V-shape at the start of fusion. They eventually fuse into an almost heart-shaped embryo with further aggregation and condensation. The extraembryonic area is now called the “serosa”.

**Figure 3:**
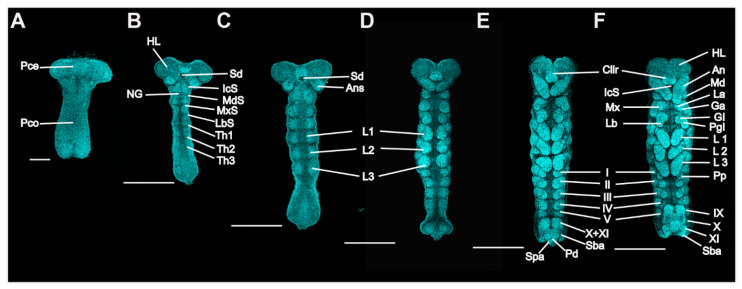
*P. americana* embryos in stages 3 to 7. Ventral views; the bent embryos were straightened. Fluorescence images with DAPI-stained nuclei are shown. (**A**) Stage 3. (**B**) Early-stage 4. (**C**) Late-stage 4. (**D**) Stage 5. (**E**) Stage 6. (**F**) Stage 7. Scale bars: 200, 500 μm. An, antenna; AnS, antennal segment; Cllr, clypeolabrum; Ga, galea; Gl, glossa; HL, head lobe; IcS, intercalary segment; La, lacinia; Lb, labium; LbS, labial segment; L1–3, prothoracic, mesothoracic, and metathoracic legs; Md, mandible; MdS, mandibular segment, Mx, maxilla; MxS, maxillary segment; NG, neural groove; Pd, proctodaeum; Pgl, paraglossa; Pp, pleuropodium; Pce, protocephalon; Pco, protocorm; Sba, subanal lobe; Sd, stomodaeum; Spa, supra-anal lobe; Th1–3, prothoracic, mesothoracic, and metathoracic segments; I–V and IX–XI, first to fifth and ninth to eleventh abdominal segments.

Stage 3. Day 4 (Figure 1C and Figure 3A): The embryo extends on the ventral surface of the egg and differentiates to form a protocephalon and protocorm. The embryo is now called the “germ band” and can be dissected out.

Stage 4. Day 5 (Figure 1D and Figure 3B,C): Early germ-band elongation. Rudiments of appendages appear in the cephalic and thoracic regions. The rudiments of the antennae differentiate first and grow faster than the other rudiments. The sizes of the rudiments of the mandibles, maxillae, labium and thoracic legs are almost equal in this stage.

**Figure 4:**
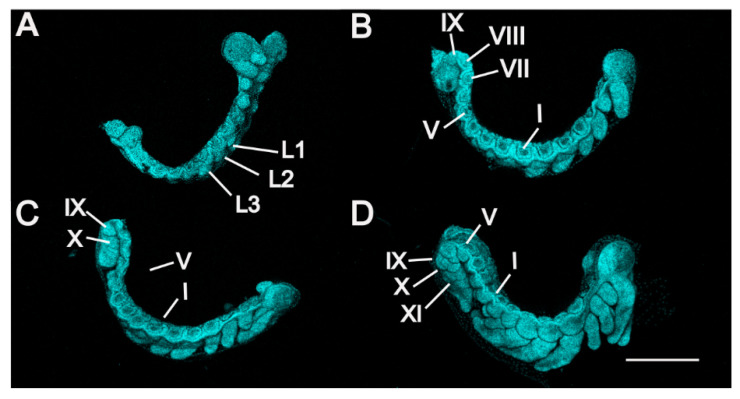
*P. americana* embryos in stages 5 to 8. Lateral views. Fluorescence images with DAPI-stained nuclei are shown. (**A**) Stage 5. (**B**) Stage 6. (**C**) Stage 7. (**D**) Stage 8. Scale bars: 500 μm. L1–3, prothoracic, mesothoracic, and metathoracic legs; I, V and VII–XI, first, fifth and seventh to eleventh abdominal segments.

Stage 5. Day 6 (Figure 1E, Figure 3D and Figure 4A): Late germ-band elongation. The germ band is further elongated along the ventral surface of the egg, and the position of its cephalic region remains almost unchanged. The proctodaeum is differentiated to form, while segmentation proceeds to the fifth abdominal segment. Related to the formation of the proctodaeum, the differentiation of the tenth and eleventh abdominal segments and the telson (anal lobes) occurs before that of the sixth to ninth abdominal segments (Figure 3D). An unpaired clypeolabrum is differentiated to form on the anterior stomodaeum.

Stage 6. Day 7 (Figure 1F, Figure 3E and Figure 4B): The subsidence of the embryo begins, and the position of the cephalic region starts to move to the bottom. Eleven abdominal segments are completed as the posterior region of the abdomen undergoes segmentation, and all segments become distinguishable (Figure 4B). The caudal end of the embryo begins to fold toward the ventral side, occurring at the eighth, ninth and tenth abdominal segments. At the cephalic end of the embryo, the clypeolabrum turns into a weakly bilobed form (Figure 3E).

Stage 7. Day 8 (Figure 1G, Figure 3F and Figure 4C): The subsidence of the embryo is complete, with the cephalic region sinking to the bottom of the egg. The caudal end folds over ventrally at the seventh, eighth and ninth abdominal segments (Figure 4C). The leg rudiments and antennae grow faster than the other appendages, such as the maxillae and labia.

Stage 8. Day 9 (Figure 1H and Figure 4D): The head of the embryo begins to move away from the bottom. Segmentation of the leg primordium begins. The caudal fold shifts to the sixth, seventh and eighth abdominal segments.

Stage 9. Day 10 (Figure 1I): The serosa shrinks, and the yolk no longer envelops the embryo.

Stage 10. Day 11 (Figure 1J): The antennae and legs grow and extend, and the segments of the legs become distinguishable. The antennae grow faster than the other appendages.

Stage 11. Day 12 (Figure 1K): The embryo body rises back to the egg’s ventral side, and the embryo’s length reaches approximately half the ventral side of the egg.

Stage 12. Day 14 (Figure 1L): The outline of the embryo appears, and the secondary dorsal organ arises. The legs are folded.

Stage 13. Day 16 (Figure 1M): The embryo grows rapidly, and the yolk is quickly enveloped by the developing body wall. At the caudal end of the egg, the yolk becomes enclosed.

Stage 14. Day 17 (Figure 1N): Completion of the dorsal closure. The yolk and the secondary dorsal organ are completely enclosed by the body wall. The growing antennae and hind legs reach the fourth abdominal segment.

Stage 15. Day 18 (Figure 1O): The outline of the eyes is forming. The antennae and hind legs extend between the fourth and fifth abdominal segments.

Stage 16. Day 20 (Figure 1P): The eyes are morphologically developed. The antennae and hind legs extend to between the fifth and sixth abdominal segments.

Stage 17. Day 23 (Figure 1Q): The pigment of the eyes is recognizable. The third segment of the mandibles is forming. The antennae and hind legs extend to the sixth abdominal segment.

Stage 18. Day 26 (Figure 1R): The pigment of the eyes is evident. The growing antennae and hind legs almost reach between the sixth and seventh abdominal segments. The mandibles have been divided into three segments.

Stage 19. Day 29 (Figure 1S): The pigmentation of the eyes becomes more noticeable. The antennae and hind legs extend to the seventh abdominal segment. The fourth segment of the mandibles is forming.

Stage 20. Day 32 (Figure 1T): The pigmentation of the eyes is distinct. The bristles on the leg surface are distinguishable. The antennae and hind legs almost extend to between the seventh and eighth abdominal segments. The mandibles are differentiated into four distinct segments.

### 3.2. Nymphal Development

**Figure 5:**
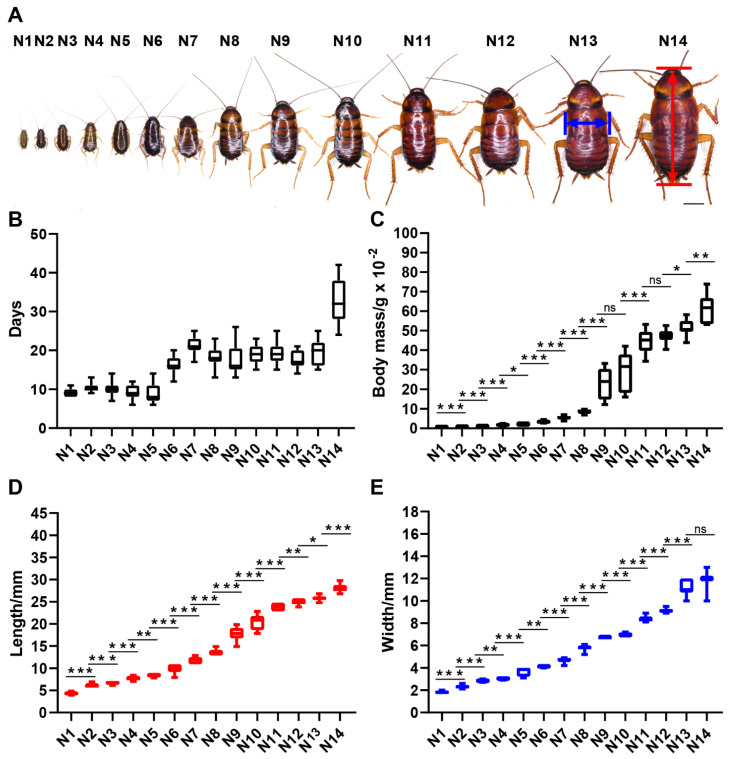
Nymphal development of *P. americana*. (**A**) Overview of the 14 nymphal instars. First-instar nymph: N1; Second-instar nymph: N2; Third-instar nymph: N3; Fourth-instar nymph: N4; Fifth-instar nymph: N5; Sixth-instar nymph: N6; Seventh-instar nymph: N7; Eighth-instar nymph: N8; Ninth-instar nymph: N9; Tenth-instar nymph: N10; Eleventh-instar nymph: N11; Twelfth-instar nymph: N12; Thirteenth-instar nymph: N13; Fourteenth-instar nymph: N14. Scale bars: 5000 μm. (**B**) The instars of the 14 nymphal instars. (**C**) The body mass of the 14 nymphal instars. (**D**) The body length of the 14 nymphal instars. (**E**) The body width of the 14 nymphal instars. ns, not significant or *p* > 0.05 between the two nymphal instars; * *p* < 0.05; ** *p* < 0.01; *** *p* < 0.001.

American cockroaches with aggressive characteristics were raised in groups. The observations showed that with sufficient living space and survival materials, nymphs of the gregarious American cockroach undergo 14 molts before transforming into adults. Development data for nymphs housed in groups are given in Figure 5 and Table S1. Body mass, body length and body width are three indicators that can distinguish each instar clearly. Almost all adjacent instars show significant differences in the three indicators. There were no significant differences in body mass between the 9th and 10th instars and between the 11th and 12th instars, while there were significant differences in the three indicators of other adjacent instar pairs. These results will be helpful for identifying the instar of nymphs of laboratory strains and will facilitate the sampling of nymphs of a specific instar.

**Figure 6:**
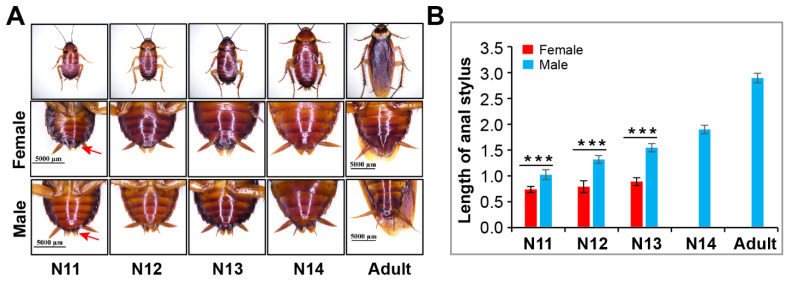
Identification of female and male of *P. americana.* (**A**) Images of female and male. Red arrows show anal stylus. (**B**) Changes in stylus length in females and males. *** *p* < 0.001. Scale bars: 5000 μm.

In the late nymphal stage, the secondary sexual characteristics of American cockroaches become distinct. As shown in Figure 6, the anal stylus of males became increasingly long beginning at the 11th instar and reached its maximum length in the adult stage, while the length of the anal stylus of females did not change significantly, and the structure disappeared at the last nymphal instar. In the 11th, 12th and 13th-instar nymphs, females had a shorter anal stylus than males.

### 3.3. Reproduction

Development of the ovary of *P. americana* occurs after eclosion of the nymph into an adult; the main features of the ovary are oocyte differentiation and vitellogenesis, mature oocytes appear on the seventh or eighth day after eclosion [21,27]. Females can produce normal oothecae whether they have mated or not. In these oothecae, both fertilized and unfertilized eggs can develop into normal nymphs, although unfertilized eggs have a lower success rate and only develop into females. This phenomenon in which unmated females can produce offspring is called parthenogenesis [28]. In contrast, when females produce offspring after mating, it is called gamogenesis.

**Figure 7:**
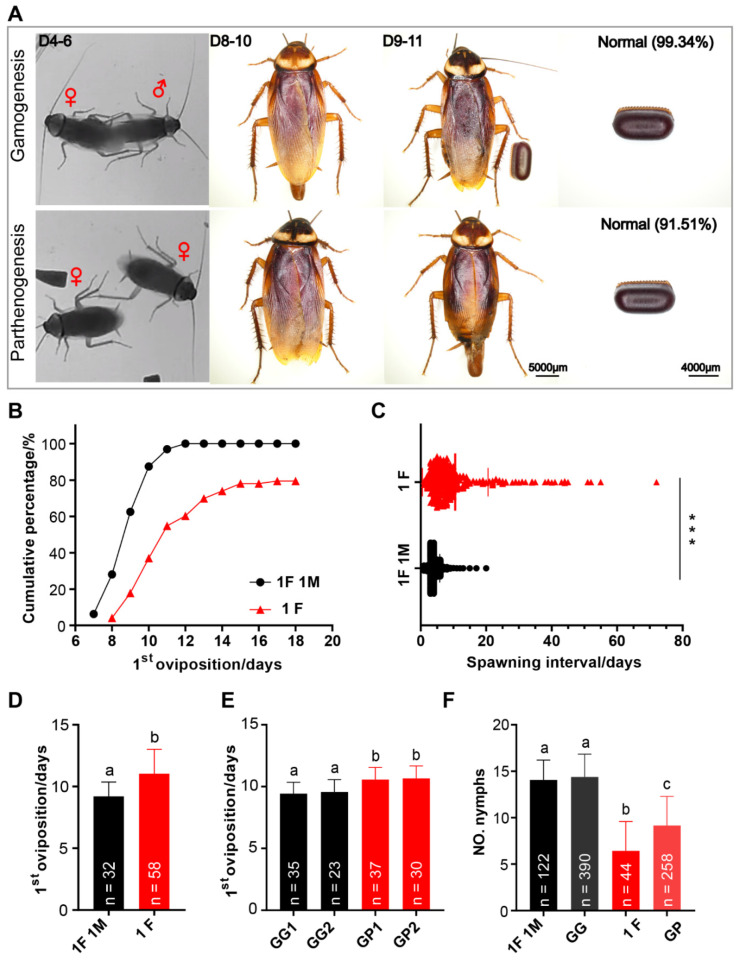
Reproduction of *P. americana*. (**A**) An overview of the first oviposition cycle. Red symbol ♀ indicates female adult and ♂ indicate male adult. Scale bars of adult: 5000 μm. Scale bars of ootheca: 4000 μm. (**B**,**D**) The time of the first oviposition while housed alone or in pairs. (**C**) The spawning interval was calculated by averaging the individual spawning intervals by unmated and mated. *** *p* < 0.001. (**E**) The time of the first oviposition while group-housed. (**F**) The number of nymphs hatching from the gamogenetic or parthenogenetic ootheca. 1F: one female; 1M, one male; GG, group-housed gamogenesis; GP, group-housed parthenogenesis; GG1, 35 females and 47 males; GG2, 23 females and 32 males. In (**D**–**F**), means with different letters are significantly different (*p* < 0.001).

Figure 7A provides an overview of the first oviposition of *P. americana*. According to our observations, when housed in pairs, female adult mate within 4–6 days after emergence (unpublished data). Among 32 biological replicates, 100% mated females laid eggs, while only 79.45% of unmated females laid eggs (73 replicates) (Figure 7B). After mating, females produced an ootheca every 4 days on average (mean ± SD = 4.10 ± 1.72) (619 data points from 29 biological replicates), and more than half of the oviposition events (64.78%) occurred 3 days or 4 days apart (Figure 7C). In contrast, unmated female adults produced an ootheca every 10 days on average (mean ± SD = 10.54 ± 10.01) (295 data points from 39 replicates), with more than half of the oviposition events (56.95%) requiring an interval of 4 to 9 days. A portion of the oothecae were randomly selected for examination, and it was found that 0.66% (N = 152) of the oothecae produced by mated females were malformed, while 8.49% (N = 106) of the oothecae produced by unmated females were malformed.

As shown in Figure 7B, the first oviposition of unmated females was delayed compared to that of mated females when housed alone or in pairs. Mated females produced the first ootheca on the 9th day after emergence on average (mean ± SD = 9.19 ± 1.18), while unmated females produced the first ootheca on the 11th day on average (mean ± SD = 11.03 ± 1.98) (Figure 7D), a significant difference. The results of group rearing were similar to those of solitary and pair rearing. When housed in groups, mated females produced their first ootheca on day 9 after eclosion on average (replicate 1: 9.43 ± 0.90, replicate 2: 9.57 ± 0.97), and that of unmated females was produced on day 10 after eclosion on average (replicate 1: 10.57 ± 0.97, replicate 2: 10.67 ± 0.98) (Figure 7E). These data suggest that when American cockroaches reproduce by parthenogenesis, approximately one additional day is required for eggs to be laid.

Observations of ootheca hatching showed a rate of 80.79% (N = 151) when the American cockroach reproduced by gamogenesis, but only 45.36% (N = 97) of the oothecae produced through parthenogenesis hatched. The oothecae produced by mated females hatched approximately 14 nymphs each (mean 1F 1M = 14.07 ± 2.13, mean GG = 14.39 ± 2.44), and there was no significant difference between group rearing and pair rearing (*p* = 0.1929 > 0.05) (Figure 7F). The average numbers of hatched nymphs from oothecae produced by solitary unmated females and group-housed unmated females were 6 (M + SD = 6.43 ± 3.11) and 9 (M + SD = 9.15 ± 3.13), respectively; there was a highly significant difference between group rearing and solitary rearing (*p* = 2.06847 × 10^−7^ < 0.001).

## 4. Discussion

### 4.1. Formation of the Embryo

Although the embryonic development of American cockroaches has been described before, the observation results were limited by the available techniques used [29]. Using staining with the fluorescent dye DAPI and confocal microscopy imaging, we obtained a more comprehensive picture of embryonic development, including the formation of the embryo, which had not been described before. On the posterior ventral side of the egg, a pair of blastoderm regions with higher cellular density in the lateral areas migrate medially and then fuse into an almost heart-shaped embryo. This type of embryo formation is also found in other insects in Polyneoptera, such as *E. yasumatsui* [26], *Gryllus bimaculatus* [30,31,32], *Galloisiana yuasai* [33] and *Zorotypus caudelli* [34]. This observation provides evidence for the hypothesis that embryo formation with the fusion of paired areas of higher cellular density is a potential autapomorphy of Polyneoptera.

### 4.2. Variability in the Number of Nymphal Instars

The American cockroach has a long developmental period, and its nymphal period ranges from 134 days to 1031 days [3]. The large differences in nymphal stage timing may be related to changes in the number of nymphal instars. Previous research results showed that the number of nymphal instars in the American cockroach is variable, ranging from 9 to 14 [18,19]. Such intraspecific variation in nymphal instar numbers is widespread across insect taxa and is found in most major orders, including those of hemimetabolous and holometabolous insects [20]. For example, in Blattaria, the numbers of nymphal instars in cockroaches, including *B. germanica* [18,35,36,37,38,39], *B. asahinai* [24], *Diploptera punctata* [18,40,41] and *Periplaneta japonica* [42], are variable. This intraspecific variability in the number of nymphal instars is influenced by factors such as temperature, photoperiod, humidity, nutrition, population density, genetics, and sex [20].

In our study, the American cockroach had 14 nymphal instars under conditions of sufficient food, water and living space. However, the statistical results for the body weight, length and width of the 14 nymphal instars showed that not all adjacent instar pairs had extremely significant differences (*p* < 0.001) in these three indicators (Figure 5; Table S2), such as between N4 and N5, N5 and N6, N9 and N10, N11 and N12, N12 and N13, N13 and N14, and even N11 and N13. Perhaps these nymphal instars are key stages determining the variation in nymphal instar numbers of the American cockroach.

### 4.3. Group Living and Reproduction

Group living can be found in almost all animal taxa because social life provides benefits to group members [43]. For example, housing cockroaches in groups promoted nymphal development and decreased the mortality rate [44,45,46,47], and the synthesis of juvenile hormone and oocyte maturation were accelerated [48]. Moreover, the effect of group living is exerted not only on the reproductive mode but also the parthenogenic mode of females [49]. When housed in groups, unmated females tended to produce parthenogenetic oothecae earlier than those housed alone.

Our study revealed that the parthenogenetic oothecae of group-housed females yielded more nymphs than those of solitary females (Figure 6F). This suggests that group living can improve the success rate of parthenogenesis in American cockroaches. Furthermore, we confirmed that the first oviposition of unmated females was delayed compared to that of mated females under both group living and solitary living. Mated females lay their first ootheca approximately one day earlier than unmated females. The earlier oviposition of mated females may be related to mating behavior and male secretions such as sex peptides and sperm [50,51].

### 4.4. The Lifespan of Adults

The adult lifespan is an integral part of the life history of cockroaches. There are several studies that describe this process. At room temperature (25–30 °C) (IN, USA), the lifespan of adult females varied from 102 to 588 days, with an average of 450 days [52]. At 29 °C to 37 °C (HI, USA), the average lifespan of mated adult females was 311 days, and the average lifespan of unmated females was 397 days [11]. When the temperature was maintained at 30 °C, the average lifespan of mated adult females was 187 days, and the average lifespan of unmated females was 212 days [12]. The lifespan data could further increase our understanding of the American cockroach.

## 5. Conclusions

This study provided a life table for *P. americana* under laboratory conditions at 29 °C. There were 14 nymphal instars of the American cockroach when housed in groups with sufficient living space and survival materials. Observations of embryonic development showed that the formation of embryos in the American cockroach is similar to that in other insects in Polyneoptera. The gamogenetic females produced their first ootheca earlier than those that underwent parthenogenesis, and the oviposition rate was higher than that with parthenogenesis throughout adulthood. Surprisingly, group living seems to improve the survival rate of parthenogenetic American cockroaches.

## Data Availability

Data is contained within the article or supplementary material.

## Supplementary Materials

The following supporting information can be downloaded at: https://www.mdpi.com/article/10.3390/insects13060551/s1, Table S1: Mean values of nymph instar, body weight, body length and body width; Table S2: *p* values of body weight, body length, and body width between adjacent pairs of nymphal instars.
