# Supplementary material for: Life-History Traits from Embryonic Development to Reproduction in the American Cockroach
Source: Insects. 2022 Jun 16;13(6):551. doi: 10.3390/insects13060551 (PMC9225176; doi:10.3390/insects13060551)
Supplement: Supplementary file 1 [file insects-13-00551-s001.zip › insects-1723675-supplementary.pdf]

Table S1. Mean values of nymph instar, body weight, body length and body width.

Nymph	Instar (days)		Body weight (g x 10^{-2})		Body length (mm)		Body width (mm)	
	Mean ± SD	N	Mean ± SD	N	Mean ± SD	N	Mean ± SD	N
N1	9.13 ± 0.94	1627	0.40 ± 0.02	10	4.39 ± 0.30	10	1.82 ± 0.11	10
N2	10.43 ± 1.02	741	0.58 ± 0.06	10	6.21 ± 0.34	10	2.31 ± 0.15	10
N3	9.83 ± 1.65	374	0.91 ± 0.12	10	6.76 ± 0.28	10	2.84 ± 0.10	10
N4	9.23 ± 1.62	191	1.78 ± 0.48	10	7.80 ± 0.51	12	3.03 ± 0.08	10
N5	9.09 ± 2.53	93	2.16 ± 0.20	10	8.49 ± 0.49	12	3.68 ± 0.37	10
N6	16.28 ± 1.83	39	3.43 ± 0.60	10	10.06 ± 0.97	12	4.11 ± 0.07	10
N7	21.07 ± 1.90	73	5.37 ± 1.06	10	11.91 ± 0.85	10	4.67 ± 0.24	10
N8	18.00 ± 2.29	51	8.55 ± 1.06	10	13.70 ± 0.82	10	5.80 ± 0.26	10
N9	17.56 ± 3.83	41	23.49 ± 7.56	14	18.04 ± 1.57	14	6.77 ± 0.09	10
N10	19.22 ± 2.27	32	29.27 ± 9.19	14	20.57 ± 1.91	14	6.98 ± 0.10	11
N11	19.67 ± 2.73	36	44.35 ± 5.71	11	24.00 ± 0.89	11	8.40 ± 0.27	11
N12	17.79 ± 2.41	28	44.42 ± 3.67	11	25.20 ± 0.63	10	9.11 ± 0.17	10
N13	19.59 ± 3.15	27	51.44 ± 4.09	10	26.00 ± 0.67	10	11.20 ± 0.79	10
N14	32.85 ± 5.64	27	61.75 ± 7.39	10	28.30 ± 0.95	10	11.90 ± 0.88	10

* Nymphal instars were the result of multiple observations, and only those groups with a population of 10 or more were counted.

Table S2. P values of body weight, body length, and body width between adjacent pairs of nymphal instars. (A) Body weight. (B) Body length. (C) Body width.

Nymph	N1	N2	N3	N4	N5	N6	N7	N8	N9	N10	N11	N12	N13
N2	2.92×10^{-8}												
N3		2.60×10^{-7}											
N4			2.94×10^{-5}										
N5				0.031636									
N6				2.21×10^{-6}	5.27×10^{-6}								
N7						8.34×10^{-5}							
N8							2.67×10^{-6}						
N9								3.33×10^{-6}					
N10									0.080525				
N11										8.55×10^{-5}			
N12											0.148923		
N13											0.004292	0.028303	
N14											7.66×10^{-6}		0.001144

* (A) P values of body weight.

Nymph	N1	N2	N3	N4	N5	N6	N7	N8	N9	N10	N11	N12	N13
N2	2.29×10^{-10}												
N3		9.58×10^{-4}											
N4			1.16×10^{-5}										
N5				0.002545									
N6				3.88×10^{-7}	5.52×10^{-5}								
N7						1.35×10^{-4}							
N8							1.44×10^{-4}						
N9								6.78×10^{-8}					
N10									7.22×10^{-4}				
N11										1.42×10^{-5}			
N12											0.002316		
N13											1.51×10^{-5}	0.013089	
N14											1.78×10^{-9}		6.48×10^{-6}

* (B) P values of body length.

Nymph	N1	N2	N3	N4	N5	N6	N7	N8	N9	N10	N11	N12	N13
N2	1.87×10^{-7}												
N3		2.74×10^{-8}											
N4			1.66×10^{-4}										
N5				3.49×10^{-5}									
N6				4.78×10^{-17}	0.001928								
N7						1.45×10^{-6}							
N8							7.37×10^{-9}						
N9								1.63×10^{-9}					
N10									7.64×10^{-5}				
N11										5.36×10^{-13}			
N12											9.00×10^{-7}		
N13											9.64×10^{-10}	1.73×10^{-7}	
N14											1.08×10^{-10}		0.076638

* (C) P values of body width.
